# Structural Analysis of Collagen Type I Interactions with Human Fibronectin Reveals a Cooperative Binding Mode

**DOI:** 10.1074/jbc.M113.469841

**Published:** 2013-05-06

**Authors:** Michèle C. Erat, Barbara Sladek, Iain D. Campbell, Ioannis Vakonakis

**Affiliations:** From the Department of Biochemistry, University of Oxford, Oxford OX1 3QU, United Kingdom

**Keywords:** Collagen, Extracellular Matrix Proteins, Fibronectin, NMR, X-ray Crystallography, GBD, SAXS

## Abstract

Despite its biological importance, the interaction between fibronectin (FN) and collagen, two abundant and crucial tissue components, has not been well characterized on a structural level. Here, we analyzed the four interactions formed between epitopes of collagen type I and the collagen-binding fragment (gelatin-binding domain (GBD)) of human FN using solution NMR, fluorescence, and small angle x-ray scattering methods. Collagen association with FN modules ^8–9^FnI occurs through a conserved structural mechanism but exhibits a 400-fold disparity in affinity between collagen sites. This disparity is reduced in the full-length GBD, as ^6^FnI^1–2^FnII^7^FnI binds a specific collagen epitope next to the weakest ^8–9^FnI-binding site. The cooperative engagement of all GBD modules with collagen results in four broadly equipotent FN-collagen interaction sites. Collagen association stabilizes a distinct monomeric GBD conformation in solution, giving further evidence to the view that FN fragments form well defined functional and structural units.

## Teaser

**Background:** The fibronectin (FN)-collagen interaction is important for cell adhesion and migration.

**Results:** FN modules ^8–9^FnI interact with two distinct sites in both chains of collagen I. All six collagen-binding FN modules interact cooperatively with a single collagen site.

**Conclusion:** Collagen I possesses four equipotent sites for FN.

**Significance:** We have mapped FN binding to collagen I and demonstrated the first cooperative interaction.

## Introduction

Fibronectin (FN)^3^ and collagen, two essential components of the extracellular matrix, are key players in diverse cellular processes, including adhesion, migration, growth, and differentiation (1, 2). FN is a high molecular weight multidomain protein composed of three conserved module types (I, II, and III), individual structures of which have been elucidated previously (3). Biophysical studies initially suggested that FN modules are arranged sequentially, similar to beads on a string (4–6). However, more recent studies have shown the presence of compact multidomain units in FN (7–10), including the six FN modules that are important for binding to collagen (^6^FnI^1–2^FnII^7–9^FnI) (7, 8, 11–13).

The FN-collagen interaction is well documented (14), but its molecular details have remained elusive until recently. It has long been known that FN is crucial for fibroblast attachment to collagen matrices (15, 16) and for organization of collagen type I fibrils (17). *In vitro*, however, ^6^FnI^1–2^FnII^7–9^FnI binds strongly to gelatin, the denatured form of collagen (18, 19), but not to triple-helical collagen fibrils; ^6^FnI^1–2^FnII^7–9^FnI was thus named the “gelatin-binding domain” (GBD). To reconcile the *in vitro* and cellular findings, it was suggested that the physiological function of the FN-collagen interaction is related to clearance of denatured collagenous material during wound repair (20, 21) and binding of exposed single collagen chains (15) following fiber processing by matrix metalloproteinases during tissue growth (22). However, recent work suggested that the collagen triple helix unfolds locally at physiological temperatures (23–25), which suggested the possibility that FN could also interact with unwound collagen in intact fibers.

Previous work from our laboratory revealed that FN binds tightly to a consensus sequence on D-period 4 of the collagen type I α_1_ and α_2_ chains (26), just C-terminal of the MMP-1 cleavage site (27). The crystallographic structure of the complex between an α_1_ peptide from this site and ^8–9^FnI revealed that the collagen peptide extends the ^8^FnI antiparallel β-sheet by one strand (26), reminiscent of proteins from pathogenic bacteria bound to FnI modules (28, 29). Furthermore, we demonstrated that ^8–9^FnI can unwind triple-helical peptides from the same site in a concentration dependent manner (26).

What is the role of the remaining GBD modules? We recently proposed a composite GBD model from the isolated crystallographic structures of ^6^FnI^1–2^FnII^7^FnI and ^8–9^FnI (7) and suggested that a suitably long collagen peptide could bind cooperatively to these two GBD subfragments, thereby offering better affinity compared with isolated ^8–9^FnI binding (26). This model was markedly different from a crystal structure of the GBD in the presence of millimolar concentrations of Zn^2+^, which showed a dimeric conformation that impaired collagen binding (30). Here, we show that four collagen type I sites bind the GBD with broadly similar affinities, although only one displays a cooperative interaction involving all GBD modules. Ensemble analysis of small angle x-ray scattering (SAXS) data showed that the GBD adopts a monomeric conformation in solution, which is further stabilized by collagen peptide binding. Our findings demonstrate how FN fragments form unique functionally competent multidomain units, allowing FN to act as a versatile protein interaction hub in the extracellular matrix (31).

## EXPERIMENTAL PROCEDURES

### 

#### 

##### Material Production and Purification

FN fragments corresponding to residues 305–608 (GBD), 305–515 (^6^FnI^1–2^FnII^7^FnI), and 516–608 (^8–9^FnI) and bearing single amino acid substitutions to improve solubility and protein yields (H307D, N528Q, and R534K) were produced as described previously (7, 26, 32). Synthetic collagen peptides were purchased from GL Biochem (Shanghai, China); their sequences are provided in Table 1, and unless fluorescently tagged, they included a C-terminal tyrosine residue for UV determination of peptide concentration. Fluorescent peptides had 5-carboxyfluorescein attached to the N-terminal amine group.

**TABLE 1:** ***K_D_* values for collagen I peptide binding to FN fragments** α_1_ and α_2_ chain numbering is taken to begin at the estimated start of the helical region. “O” in peptide sequences denotes 4-hydroxyproline. NMR, ^1^H-^15^N heteronuclear single quantum correlation NMR titrations; FA, fluorescence polarization titrations using N-terminal 5-carboxyfluorescein labeling. In titrations where no binding was detected, we typically exceeded 2 mm in peptide concentration.

	*K_D_*			Method	Name
	GBD	^8–9^FnI	^6^FnI^1–2^FnII^7^FnI		
**Collagen type I α_1_ peptide**					
G^70^PQGARGLOGTAGLOGMKGHRGFSGLDGAKGDAGPAGPKGEOGSOGENG^118^	98 ± 8 μm			FA	A
G^76^LOGTAGLOGMKGHRGFSGLDG^97^-Y		143 ± 17 μm		NMR	A_N_
G^91^FSGLDGAKGDAGPAGPKGEOGSOGEN^117^-Y*^a^*			No binding	NMR	A_C_
G^772^PQGIAGQRGVVGLOGQRGERGFOGLOGPSGEOGKQGPSGASGERGPOG^820^	15 ± 2 μm			FA	B
G^775^LOGQRGVVGLOGQRGERGFOGLOG^799^-Y		5 ± 1 μm*^b^*		NMR	B_N_
G^778^QRG**S**VGLOGQRGERGFOGLOG^799^-Y		Slow NMR time scale interaction		NMR	
G^778^QRGV**S**GLOGQRGERGFOGLOG^799^-Y		97 ± 11 μm		NMR	
G^796^LOGPSGEOGKQGPSGASGER^816^-Y			No binding*^b^*	NMR	B_C_
**Collagen type I α_2_ peptide**					
Q^72^GARGFOGTOGLOGFKGIRGHNGLDGLKGQOGAOGVKGEOGAOGENG^118^	26 ± 3 μm			FA	C
G^76^FOGTOGLOGFKGIRGHNGLDG^97^-Y		2.0 ± 0.2 mm		NMR	C_N_
G^91^HNGLDGLKGQOGAOGVKGEOGAOGENG^118^-Y*^a^*			248 ± 12 μm	NMR	C_C_
G^772^PQGLLGAOGILGLOGSRGERGLOGVAGAVGEPGPLGIAGPOGARGPOG^820^	6 ± 1.0 μm			FA	D
G^778^AOGILGLOGSRGERGLOGVAG^799^-Y		8 ± 2 μm*^b^*		NMR	D_N_
G^793^LOGVAGAVGEOGPLGIAGPOGARGPOG^820^-Y			No binding*^b^*	NMR	D_C_

*^a^* D. Bihan and R. W. Farndale, unpublished data.

*^b^* Published in Ref. 26.

##### NMR Spectroscopy

NMR spectrometers used superconducting magnets (Oxford Instruments) at 950- and 500-MHz proton resonance frequencies (home-built or Bruker AVANCE II consoles and room temperature or cryogenic probe heads, respectively). Spectra were recorded in PBS (20 mm Na_2_HPO_4_ (pH 7.2) and 150 mm NaCl) with 1% 4,4-dimethyl-4-silapentane-1-sulfonic acid as a calibration standard. Experiment temperatures were optimized to avoid resonance broadening due to intermediate exchange phenomena and corresponded to 25 °C (^8–9^FnI) or 37 °C (^6^FnI^1–2^FnII^7^FnI). Sequential chemical shift assignments were performed earlier (7, 26). Analysis of spectral perturbations upon protein interactions and determination of equilibrium parameters were performed as described (33).

##### Fluorescence Polarization Experiments

Fluorescence polarization measurements were performed at 25 °C in PBS using SpectraMax M5 (Molecular Devices) and PHERAstar FS (BMG Labtech) fluorometers. Samples of 75 nm labeled peptide and increasing concentrations of protein in 96-well plates were excited at 485 nm with a 515-nm cutoff, and fluorescence was observed at 538 nm. Differences in fluorescence polarization were fit using a single binding model in the program Origin (OriginLab) (33).

##### X-ray Crystallography

Crystals of the ^8–9^FnI-A_N_ collagen peptide complex were formed using the vapor diffusion method from sitting drops dispensed by a mosquito® Crystal robot (TPP Labtech). The drops consisted of 100 nl of an equimolar mixture of protein (15 mg/ml) and peptide A_N_ in 10 mm HEPES and 50 mm NaCl (pH 7.0) and 100 nl of reservoir solution containing 4.3 m NaCl and 0.1 m HEPES (pH 7.5). Crystals formed after 3 weeks at 20 °C. They were cryoprotected by transfer to reservoir solution supplemented with 25% (v/v) glycerol and flash-cooled. Data were collected at a resolution of 2.6 Å at beamline ID29 of the European Synchrotron Radiation Facility (ESRF, Grenoble, France). Data were integrated with MOSFLM (34) and scaled with Scala (35). The structure was solved by molecular replacement using Phaser (36) and one ^8–9^FnI copy (Protein Data Bank code 3EJH) as a search model. Refinement was performed in PHENIX (37) using non-crystallographic symmetry restraints between parts of chains A and B (^8–9^FnI) and chains E and F (collagen peptide) of the complex and TLS refinement with one group per FnI domain or polypeptide chain. Manual model building was performed in Coot (38). Water positions were manually identified from the electron density map in Coot. Interactions between ^8–9^FnI and the collagen peptide were analyzed using PDBePISA service from the European Bioinformatics Institute (39).

##### SAXS Data Collection and Analysis

SAXS data were collected at BioSAXS beamline X33 of the Doris storage ring at the Deutsches Elektronen-Synchrotron (DESY, Hamburg, Germany) at 20 °C and 0.15-nm wavelength. Samples in PBS were checked for monodispersity using dynamic light scattering prior to data collection. The GBD was measured at 3, 2, and 1 mg/ml concentrations. The complex of the GBD with collagen peptide C was measured at a 1:1 molar ratio using 42 μm (1.5 mg/ml) GBD. The collagen peptide C concentration was verified using an on-site refractometer. All samples were supplemented with 1 mm dithiothreitol just prior to data collection to avoid radiation damage. A fresh sample of BSA was measured as a standard. Buffer subtraction, intensity normalization, and data merging for the different sample concentrations were performed using PRIMUS (40). The radii of gyration (*R_g_*) were calculated with the AutoRg subroutine in PRIMUS, whereas *D*_max_ values were calculated using autoGNOM (41). Determination of molecular model ensembles that best fit the SAXS data was performed using the ensemble optimization method (42). Quaternary structure modeling was done with SASREF (43) using the crystal structures of ^6^FnI^1–2^FnII^7^FnI (Protein Data Bank code 3MQL) (7) and one copy of ^8–9^FnI (code 3EJH) (26). Back-calculation of scattering curves from known crystal structures was performed using CRYSOL (44).

##### Miscellaneous

Collagen type I α_1_ and α_2_ numbering is taken to begin at the estimated start of the triple-helical region. This is equivalent to numbering in the UniProt database minus 178 residues for α_1_ and 90 residues for α_2_. “O” in peptide sequences denotes 4-hydroxyproline. FN residues correspond to UniProt entry B7ZLF0. The ^8–9^FnI-B_N_ crystallographic model and data have been deposited in the Protein Data Bank (code 3GXE).

## RESULTS

### 

#### 

##### The GBD Is an Elongated Monomer in Solution

**FIGURE 1.**
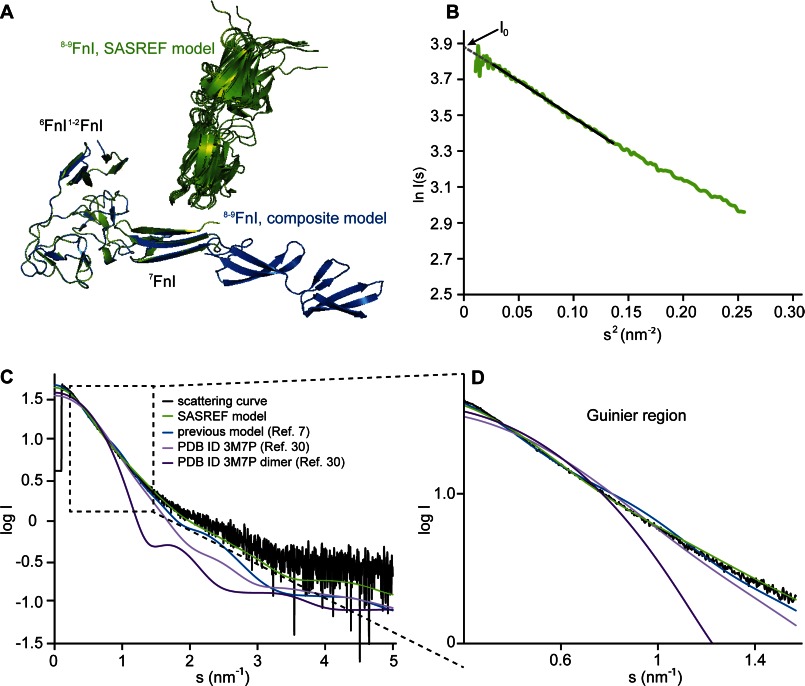
**SAXS data and GBD structure.** *A*, the previously suggested model of a monomeric GBD (*blue*) based on the crystal structures of ^8–9^FnI and ^6^FnI^1–2^FnII^7^FnI (7) compared with 10 SASREF-derived GBD models (*green*). SAXS analysis suggested an ∼90° kink between ^7^FnI and ^8^FnI. *B*, Guinier analysis of the SAXS curve for the GBD yielded an *R_g_* of 3.45 nm and an *I*_0_ of 48.77. *C*, scattering curve of 3 mg/ml GBD overlaid with back-calculated CRYSOL curves from the previously proposed composite GBD model (*blue*) (7), monomeric and dimeric versions of the GBD crystal structure (*dark* and *light purple*) (30), or the GBD SASREF model (*green*). *D*, As expected, the SASREF model fits the measured data best, especially in the crucial low angle region.

Previously, NMR solution data indicated that FN modules do not undergo radical structural rearrangement in the complete GBD compared with its subfragments ^6^FnI^1–2^FnII^7^FnI and ^8–9^FnI (7, 26). We therefore proposed a GBD model composed of the two subfragment crystal structures, with an elongated linear arrangement of ^7–9^FnI protruding from the globular ^6^FnI^1–2^FnII (Fig. 1*A*) (7). To test this model, we performed solution SAXS measurements. Three different concentrations of GBD at 3, 2, and 1 mg/ml yielded consistent scattering curves without any signs of aggregation (data not shown). Because of the higher signal/noise ratio, all further analysis was carried out with the data from the most concentrated sample using the ATSAS software package (45). Guinier analysis suggested a *R_g_* of 3.45 nm and a zero angle intensity (*I*_0_) of 48.77 (Fig. 1*B*). Using BSA as a standard, we calculated a particle molecular mass of 43 kDa, which is within the method error range for a monomeric 35.2-kDa GBD.

**FIGURE 2.**
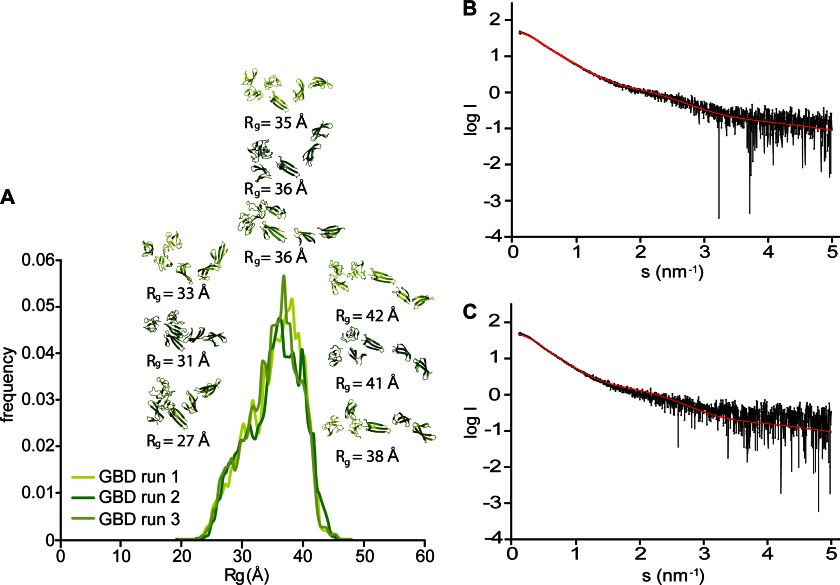
**Ensemble optimization analysis of the GBD.** *A*, three independent ensemble optimization method runs of the GBD SAXS data yielded essentially the same distribution, with an average *R_g_* centered at ∼36 Å. At 2 S.D., the width of the *R_g_* distribution of the GBD alone is 17 Å. Sample GBD models corresponding to the center and tail ends of the distribution for all three runs are shown. *B* and *C*, representative back-calculated scattering curves for the best ensembles of the GBD alone (*B*) and in a 1:1 molar complex with peptide C (*C*) compared with the respective experimental data. χ values are 0.802 (*B*) and 0.956 (*C*). χ values below 1 indicate an acceptable fit to the data.

We then used CRYSOL (44) to back-calculate the scattering curve from the composite GBD model, as well as monomeric and dimeric variants of the published GBD crystal structure (30) (Fig. 1, *C* and *D*). Comparison of the predicted scattering with the experimental data strongly favors the composite model, especially in the low angle part of the curve (χ = 2.2 *versus* χ = 6.7 for the crystallographic dimer and χ = 4.2 for the equivalent monomer). However, even our previous composite model does not adequately describe the GBD solution state as judged from the divergence of predicted and experimental scattering at high angles. Ensemble optimization analysis (42) of the scattering data yielded a broad distribution of GBD conformations with a major population cluster at *R_g_* of 3.5–3.6 nm for three independent runs, thereby confirming that this FN fragment has a unique albeit somewhat dynamic conformation in solution (Fig. 2, *A* and *B*). We used SASREF (43) to model this GBD conformation starting from either the crystallographic structures of ^6^FnI^1–2^FnII^7^FnI and ^8–9^FnI or those of ^6^FnI^1–2^FnII, ^7^FnI, and ^8–9^FnI separately. Independent runs from both inputs yielded a highly similar kinked model with an ∼90° angle between ^7^FnI and ^8^FnI (Fig. 1*A*). Interestingly, ^7^FnI is stably connected to the ^6^FnI^1–2^FnII core, as shown previously in the context of the ^6^FnI^1–2^FnII^7^FnI crystal structure (7). As expected, the back-calculated curve of this kinked model now fits the solution scattering curve much better (χ = 1.45) compared with the initial elongated model (χ = 2.2) (Fig. 1, *C* and *D*).

In summary, SAXS data are consistent with a GBD that is a monomeric in solution, with ^6^FnI^1–2^FnII^7^FnI forming a globular particle and a 90° kink between ^7^FnI and ^8^FnI. Motions around this kink likely account for the somewhat broad distribution of particle sizes in solution.

##### ^8–9^FnI Interacts with Four Sites on Collagen Type I via a Conserved Binding Mode

**FIGURE 3.**
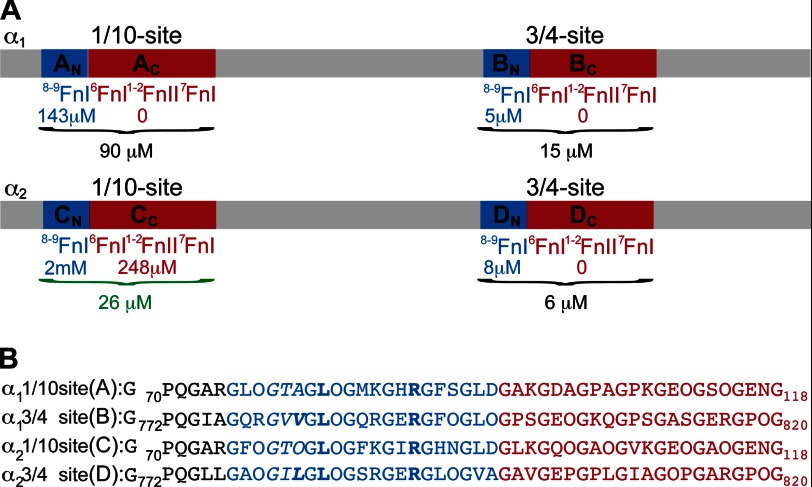
**GBD-binding sites on collagen type I.** *A*, schematic representation of the collagen type I α_1_ and α_2_ chains and the two FN-binding sites at 1/10 and 3/4 sequence distance from the collagen N terminus. ^8–9^FnI-binding sites (peptides A_N_, B_N_, C_N_, and D_N_) are shown in *blue*, with the sequences immediately C-terminal thereof (peptides A_C_, B_C_, C_C_, and D_C_) shown in *red*. Dissociation constants are indicated. Highlighted in *green* is the only site where the two GBD subfragments, ^6^FnI^1–2^FnII^7^FnI and ^8–9^FnI, bind collagen type I cooperatively. *B*, amino acid sequences of peptides A–D. Conserved positions 2 (Leu) and 9 (Arg) of the ^8–9^FnI collagen-binding epitope are in shown in *boldface*; color coding is as in *A*. The hydrophobic residue-containing triplet that enhances ^8–9^FnI affinity in the 3/4 sites is indicated in *italics*, with the crucial hydrophobic residue shown in *boldface italics*.

To firmly establish the elusive molecular interplay between the GBD and the most common collagen (type I), we first need to understand the exact nature of the binding site. On the basis of the ^8–9^FnI crystal structure in complex with a collagen type I α_1_ Gly^778^–Gly^799^ peptide (B_N_; see Fig. 3*A* for a schematic representation of all collagen peptides used here) (26), we previously suggested two potential FN-binding sites on each of the type I α_1_ and α_2_ chains. At a distance of ∼1/10 (D-period 1, peptides A_N_ and C_N_) or 3/4 (D-period 4, peptides B_N_ and D_N_) from the N terminus of the triple helix, both chains contain a consensus 9-mer ^8–9^FnI-binding sequence, in which positions 2 and 9 are occupied by leucine and arginine, respectively (Fig. 3*B*). NMR titrations showed previously (26) that the two 3/4 sites bind to ^8–9^FnI with high affinity (*K_D_* = 5 and 8 μm for B_N_ and D_N_, respectively).

**FIGURE 4.**
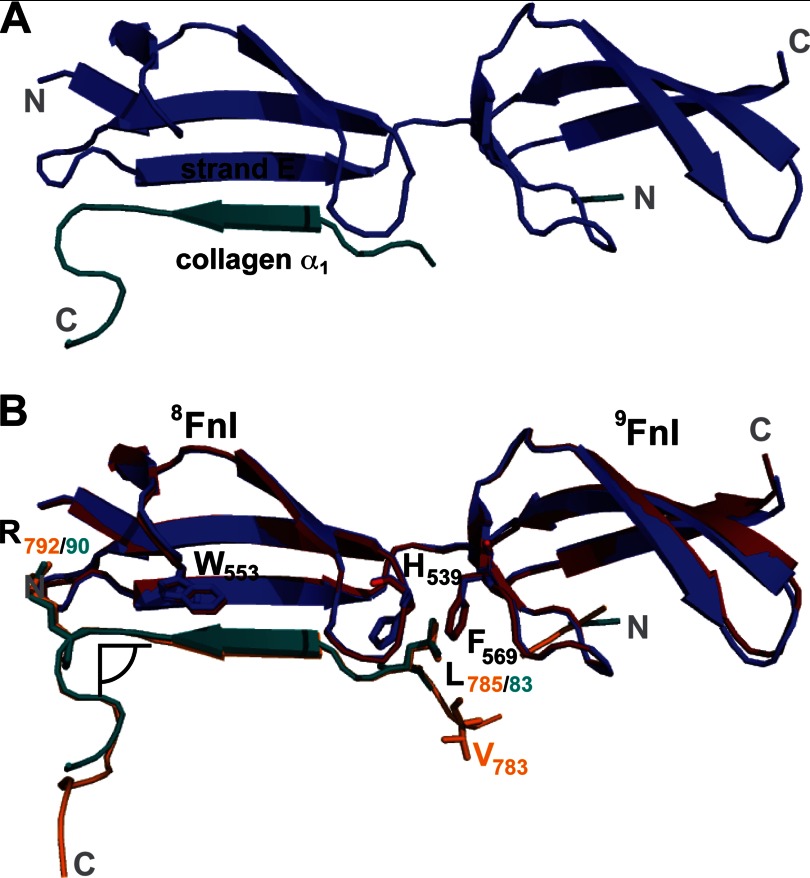
**Crystal structure of ^8–9^FnI in complex with a peptide from the collagen α_1_ 1/10 site.** *A*, schematic representation of the crystal structure of ^8–9^FnI (*blue*) in complex with the low affinity peptide A_N_ (*cyan*). *B*, overlay of the crystal structure of ^8–9^FnI (*red*) in complex with the high affinity peptide B_N_ (*orange*) (26). The antiparallel β-strand mode of binding alongside strand E of ^8^FnI is conserved, and the primary hydrophobic contacts are indicated. Val^783^, which plays an important role in increasing the affinity of peptide B_N_ for ^8–9^FnI but is not part of the consensus 9-mer sequence, is shown. A peptide hairpin just C-terminal of the consensus binding site in both collagen peptides leads to a 90° kink as indicated.

**FIGURE 5.**
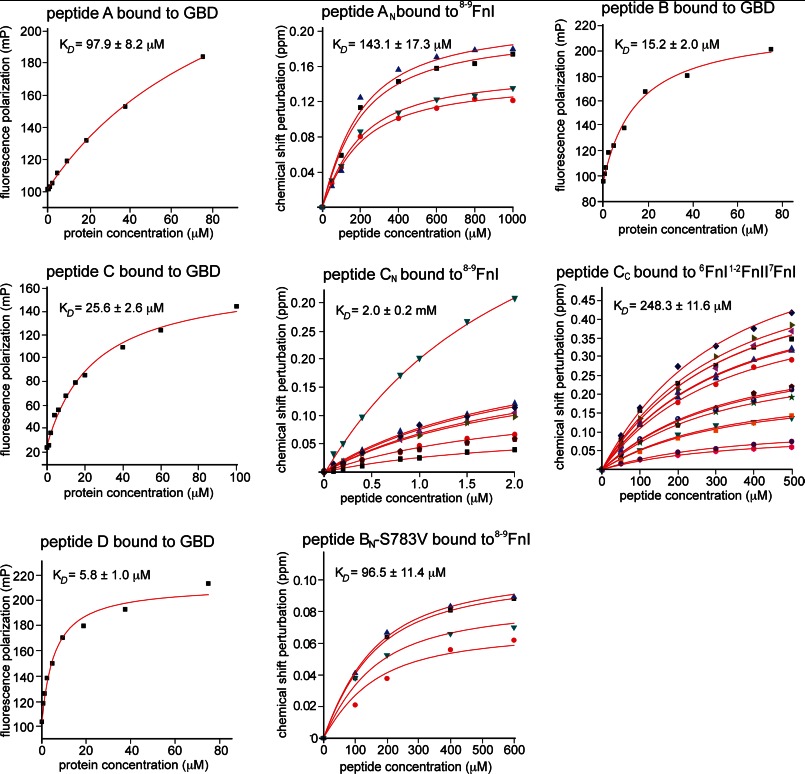
**Biophysical studies of collagen interactions with FN modules.** Shown here are the protein titration data for interactions summarized in Table 1. For NMR measurements, chemical shift perturbations are plotted against collagen peptide concentration. For fluorescence measurements, we report the polarization of peptide-bound fluorescein against the GBD concentration. All data were fit assuming a single binding event. *mP*, millipolarization units.

**FIGURE 6.**
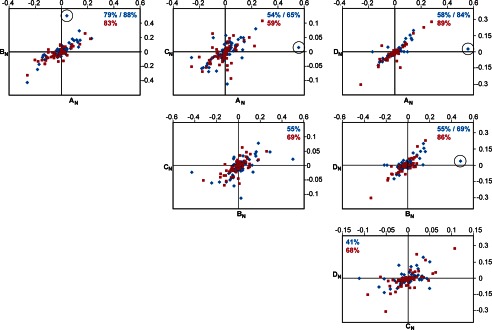
**^8–9^FnI binds collagen through a conserved interaction mode.** Shown here are pairwise comparisons of proton (*blue*) and nitrogen (*red*) chemical shift changes in ^8–9^FnI resonances upon addition of collagen peptides A_N_, B_N_, C_N_, and D_N_. Nitrogen values were divided by a factor of 5 to allow comparison with proton values on the same graph. All values are in ppm. The correlation coefficients (*R*) of independent linear fits to each data series are indicated in *blue* (for proton) or *red* (for nitrogen). In some cases, the coefficients after removal of a single proton data point (*circled*) are also shown. Note that shift changes for peptides C_N_ and D_N_ are small due to weak binding; thus, experimental errors adversely affect the correlations.

To examine the binding of a collagen 1/10 site, we determined the crystal structure of ^8–9^FnI in complex with peptide A_N_. The structure, solved to a resolution of 2.6 Å, showed A_N_ binding in an antiparallel manner to strand E of ^8^FnI (Fig. 4*A*; see Table 2 for crystallographic statistics). Thus, the molecular basis of this association is the strand extension mechanism commonly used by complexes involving FnI modules (28, 29), including the high affinity ^8–9^FnI-B_N_ complex (26). In-depth comparison of ^8–9^FnI-A_N_ with ^8–9^FnI-B_N_ revealed a striking similarity in atomic interactions. In both complexes, the indole ring of ^8^FnI Trp^553^ stacks above a glycine residue of the peptide main chain (Gly^88^ in A_N_), and an important collagen leucine (Leu^83^) is sandwiched between FN His^539^ and Phe^569^ (Fig. 4*B*). A crucial electrostatic interaction between an arginine (Arg^90^) on A_N_ and ^8^FnI Asp^516^ is also present in both structures. We were therefore surprised by NMR titrations, which revealed significantly weaker ^8–9^FnI binding affinities for the 1/10 sites (*K_D_* = 143 μm for A_N_ and 2 mm for C_N_) (Fig. 5 and Table 1) compared with their 3/4 counterparts. Comparisons of proton and nitrogen chemical shift changes in ^8–9^FnI upon addition of collagen peptides showed good correlations between the binding of high (B_N_ and D_N_) and low (A_N_ and C_N_) affinity sites (Fig. 6). These chemical shift changes report on structural perturbations induced by complex formation; thus, we conclude that the core binding mechanism of all four collagen sites for ^8–9^FnI is similar, and the reasons for the apparent differences in affinity must lie elsewhere.

**TABLE 2:** **Crystallographic data and refinement statistics** r.m.s.d., root mean square deviation.

**Data statistics**	
Cell parameters	*a* = *b* = 56.57, *c* = 152.66 Å; α = β = 90°, γ = 120°
Wavelength (Å)	0.9792
Resolution (Å)	46.68–2.6 (2.6–2.74)
Unique reflections	16,850
*R*_merge_	0.074 (0.442)
Completeness (%)	99.8 (100.0)
Multiplicity	3.4 (3.5)
*I*/σ(*I*)	14.5 (2.4)

**Refinement statistics**	
Resolution (Å)	46.6–2.6
Unique reflections	
Working set (%)	92.6
Free set (%)	7.4
*R*_work_	0.219
*R*_free_	0.271
Overall mean *B* values (Å^2^)	57.6
No. of amino acid residues/asymmetric unit (protein and ligand)	232
No. of water molecules	44
Matthews coefficient	2.68 (solvent content, 54.05%)
r.m.s.d. from ideal values	
Bonds (Å)	0.003
Angles	0.683°
Estimated overall coordinate error based on maximum likelihood (Å)	0.990
Ramachandran plot statistics (%)	
Residues in favored regions	95.9
Residues in allowed regions	4.1
Residues in disallowed regions	0.0

Just N-terminal of the consensus 9-mer sequence, both high affinity collagen 3/4 sites contain two hydrophobic residues, whereas the weaker 1/10 sites harbor a GTA (A_N_) or GTO (C_N_) triplet (Fig. 3*B*). This residue triplet makes no contacts with ^8–9^FnI in the A_N_ or B_N_ complex structure and has poor local electron density (Fig. 4*B*). Nonetheless, we reasoned that it could interact transiently with exposed hydrophobic residues of ^9^FnI, such as Phe^569^ or Ile^592^, thereby strengthening the association. To explore this hypothesis, we substituted either Val^782^ or Val^783^ in B_N_ with serine and tested for ^8–9^FnI binding using NMR titrations. Whereas substitution of Val^782^ did not alter the association significantly, maintaining a slow time scale interaction regime similar to the wild type, when Val^783^ was changed, the affinity for ^8–9^FnI dropped to ∼100 μm and a fast interaction regime. Thus, we conclude that although the ^8–9^FnI binding mode is conserved for all four collagen type I sites, residues outside the central consensus 9-mer sequence influence the association strength. This most adversely affects C_N_ at the 1/10 site of the α_2_ chain, likely due to the substitution of both hydrophobic residues in the triplet above with highly hydrophilic ones.

##### The GBD Binds Cooperatively to the Collagen α_2_ 1/10 Site

Could the remaining modules of the GBD strengthen the FN interaction with collagen? Interestingly, the collagen peptide in both ^8–9^FnI-A_N_/B_N_ crystal structures displays a 90° kink just C-terminal to the consensus ^8–9^FnI-binding sequence, stabilized by hydrophobic interactions involving the Phe^92^ (A_N_) or Phe^794^ (B_N_) side chain and the peptide main chain (Fig. 4*B*). Hydrophobic residues exist in equivalent positions in C_N_ and D_N_ as well, thus raising the possibility that this peptide kink is a common feature of all four collagen sites (Fig. 3*B*). This change in peptide direction matches well the relative orientation of ^8–9^FnI with respect to ^6^FnI^1–2^FnII^7^FnI in the SAXS-derived GBD model presented above. Indeed, when we overlay the ^8–9^FnI-A_N_/B_N_ crystal structures onto the SASREF GBD model, the collagen peptides follow the GBD kink, so their C termini would be ideally located to bind to ^6^FnI^1–2^FnII^7^FnI. These findings are consistent with our earlier suggestions of possible cooperative collagen binding to the GBD (7); thus, we explored whether extending the four collagen peptides toward their C terminus increases their affinity for the GBD compared with ^8–9^FnI.

**FIGURE 7.**
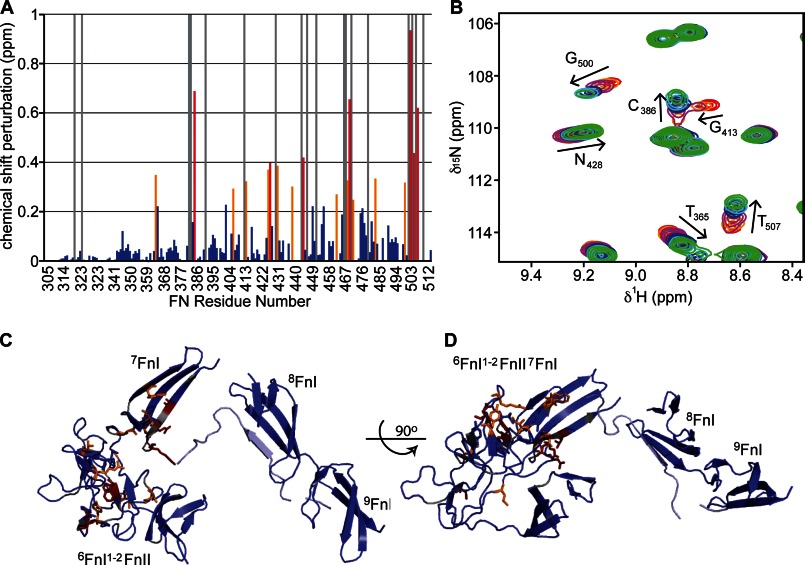
**NMR chemical shift analysis of the interaction between ^6^FnI^1–2^FnII^7^FnI and peptide C_C_.** *A*, combined amide chemical shift differences. *Red bars* indicate perturbations >2 S.D. from the mean, and *orange bars* indicate perturbations >1 S.D. *Blue bars* denote measured chemical shift perturbations < 1 S.D. *Gray bars* indicate peak disappearance upon titration with the peptide. *B*, region of a ^1^H-^15^N heteronuclear single quantum correlation NMR spectrum showing an overlay of ^6^FnI^1–2^FnII^7^FnI resonances that shift upon addition of peptide C_C_. *C* and *D*, two perpendicular representations of the GBD SASREF model with residues in stick representations and colored according to the chemical shift perturbations found in *A*. The *light blue* collagen peptide indicates how the most perturbed residues in ^2^FnII and ^7^FnI can form a continuous collagen-binding interface with ^8–9^FnI.

The long peptides A, B, and D (Fig. 3*A* and Table 1) bound to the full-length GBD with affinities comparable to ^8–9^FnI alone, as shown by fluorescence polarization experiments (Fig. 5). These results suggest that ^6^FnI^1–2^FnII^7^FnI does not add significantly to the collagen interaction once ^8–9^FnI is bound to peptides A_N_, B_N_, and D_N_. This observation was supported by NMR titrations of just the C-terminal segments of these peptides (A_C_, B_C_, and D_C_) (Fig. 3*A*) with ^6^FnI^1–2^FnII^7^FnI, where no appreciable binding was detected (Table 1). In contrast, the long peptide C, which extends from the weakest ^8–9^FnI interaction site at the 1/10 position of the α_2_ chain, interacted with the full-length GBD with *K_D_* ≈ 26 μm (Fig. 5), which represents an ∼80-fold enhancement compared with the ^8–9^FnI-C_N_ interaction alone (Table 1). The C terminus of this peptide (C_C_) bound ^6^FnI^1–2^FnII^7^FnI with an affinity of *K_D_* ∼ 250 μm, as measured by chemical shift analysis of NMR titrations (Figs. 5 and 7, *A* and *B*, and Table 1). As shown in Fig. 7 (*C* and *D*), structural perturbations upon peptide C_C_ binding to ^6^FnI^1–2^FnII^7^FnI occur over a single molecular surface that extends from the ^8–9^FnI collagen-binding interface. We conclude that peptide C, the collagen 1/10 site of the α_2_ chain, binds the full-length GBD and not just the ^8–9^FnI modules; to our knowledge, this is the first time a specific cooperative binding site for the full-length GBD has been found.

##### Collagen Binding Stabilizes the Solution GBD Conformation

**FIGURE 8.**
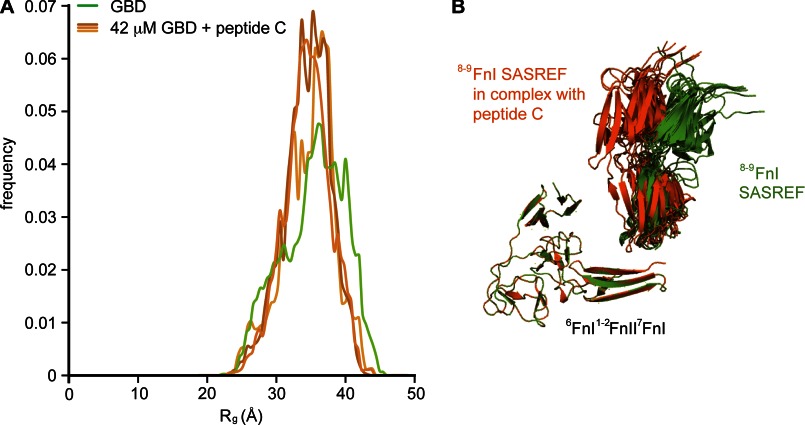
**SAXS analysis of the GBD in complex with collagen.** *A*, ensemble optimization analysis of the GBD alone or in a 1:1 complex with peptide C. Upon complex formation, the *R_g_* distribution narrows through disappearance of minor conformational states. *B*, schematic representation of 10 SASREF models of the GBD alone (*green*) or in complex with peptide C (*orange*). All structures are aligned at the ^6^FnI^1–2^FnII^7^FnI subfragment. Peptide binding does not lead to a major structural rearrangement but stabilizes the pre-existing major conformation.

To investigate the effects of the cooperative binding of collagen peptide C to the GBD structure, we measured SAXS data on this 1:1 complex and obtained scattering curves without any sign of aggregation. Three independent ensemble analysis runs of these data yielded a monodisperse distribution of molecular models, with an average *R_g_* of ∼3.5 nm (Figs. 2*C* and 8*A*), which is essentially identical to the major population cluster of the GBD alone. Although the fit is still reasonable, it deviates further than calculations of the GBD alone. We attribute this at least partly to the fact that the contribution of the peptide to the scattering curve was not taken into account.

However, upon complex formation, the breadth of possible GBD conformations narrows through disappearance of minor states. We interpret these data in terms of stabilization of a unique GBD conformation upon collagen binding without further structural rearrangements. This interpretation is supported by an ensemble of 10 SASREF models of the GBD-C_C_ complex, which is highly similar to the ensemble of the GBD alone (Fig. 8*B*). Once again, there is a 90° kink between ^7^FnI and ^8^FnI, and the model displays a slight compaction of ^8–9^FnI toward the ^6^FnI^1–2^FnII^7^FnI core. Thus, we conclude that cooperative binding of the collagen α_2_ 1/10 site (peptide C) stabilizes a major pre-existing conformation of the GBD in solution. This supports previous findings that FN modules form distinct functional units capable of presenting a unique interface to their respective binding partners (8, 31, 46).

## DISCUSSION

We have presented a model showing how the full-length GBD of FN and the most common collagen (type I) interact on a molecular scale. Collagen binding to the GBD is mediated mostly by the ^8–9^FnI subfragment, which interacts with sites on D-period 1 (1/10 site) and D-period 4 (3/4 site) of both collagen type I chains (26). All four binding sites contain a consensus 9-mer sequence with conserved leucine (position 2) and arginine (position 9) residues acting as major interaction determinants (Fig. 3*B*) (26). However, not all sites are equal in their affinity for ^8–9^FnI, with disparities as great as 400-fold between peptides C_N_ and B_N_.

Our analysis suggests that, in select cases, the remaining GBD subfragment, ^6^FnI^1–2^FnII^7^FnI, can act to reduce this disparity. It had previously been noted that this subfragment binds to gelatin and to short collagen fragments (47, 48), and it had been suggested that ^6^FnI^1–2^FnII^7^FnI can bind triple-helical collagen prior to unwinding (7). Here, we have shown that these domains can also engage a specific collagen site at the 1/10 position of the collagen α_2_ chain (peptide C_C_) and increase the overall GBD affinity of that site to levels comparable to those of sites A, B, and D. This is the first collagen interaction found to engage all GBD modules in a cooperative manner, and we speculate that the final physiological result of this additional association is the creation of four broadly equipotent FN-binding sites on collagen type I.

SAXS analysis revealed the GBD to be a relatively elongated particle in solution, the structure of which is characterized by a 90° kink between ^7^FnI and ^8^FnI (Figs. 1*A* and 8*B*). This GBD kink is matched by a similar change in direction on the collagen peptides studied, which is the result of a local hydrophobic collapse just C-terminal of the core ^8–9^FnI-binding site (Fig. 7, *C* and *D*). Together, these two features create the potential for a snug interaction between collagen and the full-length GBD, a feature exploited by collagen peptide C. GBD modeling from SAXS data of this complex yielded a very similar albeit slightly more compact model than the GBD alone (Fig. 8, *A* and *B*). Together with the narrowing of the *R_g_* distribution in the SAXS ensemble analysis, this result indicates that the GBD does not undergo any major conformational changes upon collagen binding. Rather, we propose that the FN GBD adopts in solution a well defined major conformation, which is capable and ready for functional engagement with collagen (17, 49).

## Footnotes

3 The abbreviations used are:

FN: fibronectin
GBD: gelatin-binding domain (collagen-binding subfragment of fibronectin)
SAXS: small angle x-ray scattering.
